# Supplementary material for: Purinergic Ca2+ Signaling as a Novel Mechanism of Drug Tolerance in BRAF-Mutant Melanoma
Source: Cancers (Basel). 2024 Jun 30;16(13):2426. doi: 10.3390/cancers16132426 (PMC11240618; doi:10.3390/cancers16132426)
Supplement: Supplementary file 1 [file cancers-16-02426-s001.zip › Supplementary Table S1.pdf]

Supplementary Table S1. Reagent information.

**Compound**	**Solvent**	**Vendor**
PLX4720	DMSO	MedChemExpress, HY-51424
Dabrafenib	DMSO	MedChemExpress, HY-14660
Trametinib	DMSO	MedChemExpress, HY-10999
AACBA hydrochloride	MilliQ water	Alamone Labs, A-410
AZD9056 hydrochloride	DMSO	MedChemExpress, HY-19427A
A740003	DMSO	MedChemExpress, HY-50697
BX430	DMSO	Selleck Chem, S0758
isoPPADS tetrasodium salt	MilliQ water	Tocris, 0683
Gefapixant	DMSO	Selleck Chem, S6664
